# Could bilingualism contribute to *affective* reserve in Alzheimer's?

**DOI:** 10.1002/alz70861_108205

**Published:** 2025-12-23

**Authors:** Caitlin Ware

**Affiliations:** ^1^ Université de Lorraine UR4432, Nancy France

## Abstract

**Background:**

Bilingualism has been associated with increased cognitive reserve (Bialystock 2021), yet the mechanisms driving this relationship remain unclear. Di Rosa (2024) recently coined a new concept, affective reserve, to describe individual differences in resilience to stress, pathology, and aging. Our study proposes a novel framework with the hypothesis that bilingualism could contribute to this affective aspect of reserve in Alzheimer’s. Current research often overlooks the subjective and heterogeneous nature of bilingualism—factors such as age of acquisition, language combinations, and subjective perceptions of language vary widely across individuals. Similarly, Alzheimer’s disease is a heterogeneous condition, with symptom expression differing significantly between patients.

**Method:**

We conducted a literature review on psychological factors of bilingualism, and analysed the results in light of current research on bilingual cognitive reserve in Alzheimer's.

**Result:**

We propose that bilingual reserve could be examined from another angle, integrating the psychological and affective dimensions of bilingualism. From a psychodynamic standpoint, Alzheimer’s is characterized by Ego weakening and the erosion of secondary defense mechanisms, particularly repression—a key defense against internal conflict and trauma.

**Conclusion:**

Using a second language requires the capacity to inhibit the first (Green 1998), a process that resembles repression, which involves the inhibition of unconscious representations. Emerging research supports this idea, suggesting that second language use may aid in emotional regulation (Ware & Rigaud, 2022) and facilitate therapeutic processes such as transference (Byford, 2015). These findings highlight the potential for bilingualism to serve not only as a cognitive asset but also as a psychological resource, opening new pathways for understanding the psychical benefits of multilingual experience, and contribute to the understanding of bilingual reserve in the context of Alzheimer’s disease.

